# Relationship between skin snip and Ov16 ELISA: Two diagnostic tools for onchocerciasis in a focus in Cameroon after two decades of ivermectin-based preventive chemotherapy

**DOI:** 10.1371/journal.pntd.0010380

**Published:** 2022-05-02

**Authors:** Linda Djune-Yemeli, André Domché, Hugues C. Nana-Djeunga, Cyrille Donfo-Azafack, Cedric G. Lenou-Nanga, Palmer Masumbe-Netongo, Joseph Kamgno

**Affiliations:** 1 Centre for Research on Filariasis and other Tropical Diseases (CRFilMT), Yaoundé, Cameroon; 2 Molecular Diagnosis Research Group, Biotechnology Centre-University of Yaoundé I (BTC-UY-I), Yaoundé, Cameroon; 3 Parasitology and Ecology Laboratory, Department of Animal Biology and Physiology, Faculty of Sciences, University of Yaoundé I, Yaoundé, Cameroon; 4 Department of Biochemistry, Faculty of Science, University of Yaoundé 1, Yaoundé, Cameroon; 5 Faculty of Medicine and Biomedical Sciences, University of Yaoundé I, Yaoundé, Cameroon; NIH-National Institute for Research in Tuberculosis-ICER, INDIA

## Abstract

**Background:**

Onchocerciasis elimination currently relies on repeated ivermectin-based preventive chemotherapy. Current World Health Organization’s guidelines strongly recommend, though with low evidence of certainty, the use of Ov16 serology testing in children younger than 10 years old to assess whether mass drugs administration can be safely stopped. Therefore, more evidences are needed to support the use of this marker as sero-evaluation tool. This study aimed at determining the relationship between microfilaridermia and anti-Ov16 IgG4, and their variation according to age, gender and ivermectin intake history.

**Methodology:**

A cross-sectional survey was conducted in an area where ivermectin-based MDA has been implemented since more than 20 years. A questionnaire was used to record ivermectin intake history for the last 5 years. All volunteers aged ≥2 years were tested for microfilaridermia. IgG4 antibodies against Ov16 antigen were determined using the Standard Diagnostic Ov16 IgG4 ELISA kits and the recombinant anti-Ov16 AbD19432 antibodies. Prevalences, microfilaridermia counts and IgG4 concentrations were compared with regards to age, gender and history of ivermectin intake.

**Principal findings:**

The prevalence of skin microfilariae was 23.4% (95% CI: 23.4–30.8), whereas Ov16 seroprevalence was 53.2% (95% CI: 47.9–58.4). A moderate positive percentage agreement (50.4%) and a high negative percentage agreement (69.2%) was found between skin snip and Ov16 serology in the whole population, while in children aged <10 years, the agreements were higher (positive percentage agreement: 62.6%; negative percentage agreement: 83.5%). In addition, no associations were found between ivermectin intake, Mf counts and estimated IgG4 concentration of participants. Anti-Ov16 IgG4 were higher in individuals harboring microfilariae than their negative counterparts (p<0.0001), though a negative correlation was found between skin microfilarial counts and anti-Ov16 IgG4 levels (r = -0.2400; p = 0.03). No variation in microfilarial counts according to age and gender was observed. Though positively correlated with age (r = 0.4020; p<0.0001), IgG4 was significantly different between the different age classes (p<0.0001).

**Conclusion/Significance:**

Our results revealed moderate positive and negative agreements between parasitological and immunological parameters of onchocerciasis infection after several rounds MDA. Anti-Ov16 IgG4 levels increased with age but decreased with microfilarial counts, suggesting a variation of anti-Ov16 IgG4 as a result of constant exposure and accumulation of infection. This brings evidence sustaining the use of Ov16 serology in children as evaluation tool. However, additional investigations are needed to further reshape the appropriate age range among children aged <10 years old.

## Author summary

The elimination of onchocerciasis places high demands on monitoring and evaluation. The current WHO’s guidelines recommend the use of serological test (ELISA) to determine the presence of IgG4 antibodies to the *O*. *volvulus* specific antigen Ov16 among children aged <10 years old, thought with low evidence of certainty. In this paper, we explored the relationship between anti-Ov16 IgG4 antibodies and microfilaridermia counts, and assessed their variation according to age, gender and history of ivermectin intake. Our findings revealed no variation of Mf count and IgG4 with ivermectin intake. However, we observed that anti-Ov16 IgG4 decrease with microfilaridermia counts, but an increasing trend was observed with age. This brings evidence sustaining the use of Ov16 serology testing as exposition marker in children younger than 10 years. However, children age 2 to 4 years seem to have a very low anti-Ov16 IgG4 concentration, this finding should be considered when defining the age class for seroprevalence evaluation.

## Introduction

Onchocerciasis, better known as river blindness, is a debilitating insect-borne parasitic disease caused by *Onchocerca volvulus* (*Ov*) and transmitted to humans via the bites of blackflies of the genus *Simulium*. It is estimated that 120 million individuals worldwide are at risk of *Ov* infection, with >99% residing in Sub-Saharan Africa [1]. The latest World Health Organization (WHO) estimates indicate that 20.9 million people are infected with *Ov*, 14.6 million suffering from skin disease and 1.15 million being blinded [2]. Currently, Onchocerciasis control programs rely on ivermectin (IVM)-based preventive chemotherapy (PCT) repeated on a regular basis (annual or multi-annual), with at least 80% coverage to suppress and eventually interrupt transmission [3]. To reach elimination goal, close monitoring and evaluation of programs activities are necessary. Since the traditional parasitological method of counting microfilariae (Mf) from skin biopsies is not sensitive enough when prevalence and intensity of infection are low, especially after prolonged PCT [4], WHO recommends (i) pool screening of the blackflies vector for the presence of parasite DNA, and (ii) serological surveys among children aged <10 years old for the presence of Ov16 antibodies [3, 5–7] in order to demonstrate absence of transmission and make decision of stopping/resuming MDA.

Opinions on the Ov16-based serological test have been controversial, its utilization was strongly recommended by WHO but with low certainty of evidence [3]. Indeed, it was demonstrated that this test cannot differentiate between current and past infections [8], and a number of *Ov* infected individuals have some degree of genetic restriction that prevent them from eliciting any immune response to Ov16 antigen [9]. Consequently, it is assumed that any Ov16-based serological test fail to identify ~20% of *O*. *volvulus* infected individuals [7, 9]. Finally, despite Ov16 was assumed to be more immunogenic in early pre-patent period of *Ov* infection [10], recent evidences showed that antibody response to Ov16 might take 12 to 15 months to develop, and that detectable IgG4 response to Ov16 might correlate with microfilariae release under the skin [11, 12], suggesting that the production of antibody against Ov16 might not begin during the early stages of infection when skin Mf or adult worms in nodules are not yet detectable as previously thought. Considering these limitations, it is likely that Ov16 serology does not accurately inform on onchocerciasis transmission. Despite the broad used of this serological marker, there is poor understanding of its IgG4 antibodies response, and its association with skin Mf, age, gender, and IVM intake. In this study, we explored the relationship between microfilaridermia and IgG4 antibodies against Ov16, taking into account age and gender of participants, in an area where IVM-based PCT have been distributed for >20 years [12].

## Methods

### Ethics statement

This study was approved by the Institutional Review Board of the Faculty of Medicine and Biomedical Sciences of the University of Yaoundé 1 (N° 294/UY1/FMSB/VDRC/CSD). Administrative approval was granted by the Bafia District Medical Officer. Community members were briefed on the objectives and scope of the study prior to enrolment. Written informed assents and consents, as well as parental authorizations were obtained from all the participants. All data obtained and herein reported were treated anonymously by the investigators.

### Study areas and populations

The data and samples were collected in May 2019 in five communities (Biatsota, Boyabissoumbi, Guientsing I, Nyamanga, Tsekane) of the Bafia health district (4°45′00″N, 11°14′00″E). Bafia is located in the Mbam and Inoubou Division (Centre Region), at 120 km north from Yaoundé, the political capital of Cameroon. In 2017, its population was estimated at 161,400 inhabitants, based on the data of the Ministry of Public Health [13]. The altitude of this region varies from 1,100 to 1,300 m. It is a forest-savanna transition zone, irrigated by many fast-flowing rivers including Sanaga and its tributaries, as well as the Mbam and Noun Rivers favoring the development of the Simulium, vector of onchocerciasis. The main activities of inhabitants are agriculture (mainly cocoa), fishing and sand mining.

### Study design and sample size

A cross-sectional study was undertaken in five communities of the Bafia health district to explore the relationship between microfilaridermia and IgG4 antibodies against Ov16, while accounting for age, gender and compliance of participants to IVM treatment. This study took place about ten months after the last annual IVM mass distribution. All individuals aged 2 years and over were eligible for this study. Volunteers were interviewed regarding their five-year compliance history to IVM-based MDA as well as the duration of their residency in the communities, before they undergone parasitological and serological examinations.

Considering the previously reported microfilaridermia prevalence of 24.4% [14] in the Bafia health district and its population estimated at 161,400 inhabitants, a minimal sample size of 284 individuals was needed to assess the prevalence of onchocerciasis in this population with a precision of 5% and 95% confidence. In addition, given that this study also compared two diagnostic tests, the Buderer’s formula [15] was further used to confirm that this samples size will be enough to have a confidence of 95% and a precision of 5% in comparing Ov16 ELISA to skin snip. Considering a sensitivity of 63% for skin snip microscopy, a minimum of 74 individuals were needed.

### Sample collection and processing

#### Sample collection

Skin biopsies and Dried Blood Spots (DBS) were collected as part of this study. Indeed, two skin snips were taken from each posterior iliac crest using a 2 mm corneoscleral punch (Holth-type). The skin samples were immediately placed separately into wells of microtitration plates containing a sterile normal saline solution and incubated at room temperature as previously described [16]. Also, participants were given a finger prick and resulting blood was collected on a TropBio filter paper (WHO format). After air drying, the cards were stored at 4°C in resealable plastic pouches (one per pouch) containing desiccants. DBS were returned to the Centre for Research on Filariasis and other Tropical Diseases (CRFilMT) within 09 days of collection and stored at -20°C until ELISA analysis.

#### Microscopic evaluation of skin snips

Microscopic evaluation of the skin snips for MF from the study participants was performed as previously described [16]. Indeed, after 24 hours incubation at room temperature, the fluid from each well was examined under low magnification (40×) by trained laboratory technicians. For positive results, the microfilariae were counted and the individual microfilarial densities were expressed as the arithmetic mean number of microfilariae in the two skin snips. The skin snips and Mf were then preserved separately in Isopropanol and stored at −80°C for further analysis.

#### Anti-Ov16 IgG4 ELISA and quantification of IgG4

The presence and concentration of IgG4 antibodies recognizing Ov16 antigen in DBS (containing about 10 μL of whole blood) were determined by ELISA using a commercially available Standard Diagnostic anti-Ov16 IgG4 ELISA kit (Standard Diagnostics Inc; Cat. No: 61EK11) and recombinant human anti-Ov16 IgG4 standards panel (AbD19432; lot Number: 1TER509). The recombinant anti-Ov16 IgG4 used in this assay were produced by Bio-Rad as previously described by Golden *et al*. [17]. All the controls (kit and AbD19432 Controls) and samples were diluted with a 1/50 dilution factor (5μl of control for 245μl of kit dilution buffer, and 1 DBS in 250 μl of kit dilution buffer). The assays were performed in duplicate, following manufacturer’s instruction, with slight modification brought by the Centre for Diseases Control and prevention protocol (addition of AbD19432 standard panel and quality control for validation and standardization purpose). Briefly, the DBS were eluted overnight and the following morning, samples and recombinant antibodies were exposed to a plate pre-coated with recombinant Ov16 antigens. Bound IgG4 were detected by exposure to anti-human IgG4 conjugated to horseradish peroxidase enzyme. The plates were developed with tetramethylbenzidine (TMB) substrate and absorbance read at 450nm.

For quality control, replicate OD values were used to calculate the mean OD value for each sample (including controls). The normalized OD values were calculated as the ratio of the mean OD value of a given sample to the mean OD value for the calibrator (provided by the kit). Further, the coefficient of variation (CV in %) was calculated for each sample (CV% = OD Specimen/Standard Deviation of the plate). If the CV of one of the controls (Positive control, Negative control, Calibrator or AbD19432 controls) was above 20%, the result of the entire plate was rejected and the assay was repeated. For individual samples, if the CV was above 20% and the normalized OD of the sample between 0.450–0.550, the result of that sample was rejected and repeated. The cut-off value for positivity was set at 0.500.

Recombinant AbD19432 Ab in three different concentrations (200, 50 and 25 ng/ml, diluted to 1/50) were used in each plate to estimate anti-Ov16 IgG4 antibody concentration. The mean OD for each Ab concentration was calculated for the total number of plates. Using those OD values, a calibrating curve was drawn with the concentration in Ab on x-axis and the OD value on y-axis. The estimated Ab concentration in each sample was then determined (for all the study participant) using the following formula, the dilution factor being 0.02 and the concentration expressed in ng/ml; detailed procedure of the calculation Ab concentration is provided as supplementary material (S1 Text):

$$Ab\;concentation=\frac{Sample\;OD}{Slope}*Dilution\;factor$$

### Statistical analysis

All relevant data were recorded into a Microsoft Excel spread sheet and subsequently exported to Graph pad Prism (version 6.0) for statistical analyses. Microfilaridermia and Ov16 positivity were expressed as seroprevalence or proportion with 95% confidence interval. Positive percentage agreement (PPA) and negative percentage agreement (NPA) were used to investigate the agreement between skin snip and Ov16 serology. PPA was considered as the proportion of skin Mf positive individuals with a positive Ov16 ELISA result, while NPA was considered as the proportion of skin Mf negative individuals with a negative Ov16 ELISA result. Chi-square, Mann Whitney, and Kruskal Wallis tests were used to compare seroprevalences and median skin Mf and IgG4 levels between gender and age classes, respectively. Furthermore, logistic regression was used to relate skin Mf and Ov16 prevalences to age. Finally, Spearman correlation ranked test was used to assess correlation between skin Mf and age, IgG4 levels and age and skin Mf and IgG4. The threshold for significance was set at 5% for all statistical analyses.

## Results

A total of 342 participants aged 2–86 years old (Median: 8; Interquartile range (IQR): 5–33) were enrolled in the five communities visited in the framework of this study. Females (52.1%) were most represented than males (47.9%).

### Compliance to IVM mass administration

A total of 63 (18.4%) participants were children younger than 5 years, and therefore non-eligible for IVM mass treatments. Among the 279 individuals eligible to IVM-based MDA, 166 (59.5%; 95% CI: 53.7–65.1%) reported having taken IVM at least once during the last 5 years. The compliance to IVM-based MDA for the last 5 years according to the different age classes considered in this study is given in Table 1. Overall, individuals aged 5 to 10 years had the lowest compliance while individuals older than 10 years exhibited an adhesion rate of >75%, most of them reporting having taken IVM every year during the last 5 years.

**Table 1:** History of IVM intake for the last five years according to age classes.

Age of participants	N Interviewed	Never swallowed IVM	1 round IVM	2 rounds IVM	3 rounds IVM	4 rounds IVM	5 rounds IVM
[5–10]	144	93	30	10	4	3	4
[11–20]	29	10	9	2	0	2	6
[21–30]	16	4	3	0	0	3	6
[31–40]	18	3	4	1	0	2	8
[41–50]	21	1	3	0	3	2	12
[51–60]	28	1	2	1	2	4	18
[61-over]	23	1	0	0	1	0	21
**Total (%)**	**279 (100)**	**113 (40.5)**	**51 (18.3)**	**14 (5)**	**10 (3.6)**	**16 (5.7)**	**75 (26.9)**

### Microfilaridermia prevalence

Among the 342 individuals examined, 80 (23.4%; 95%CI: 19.2–28.2) harbored *O*. *volvulus* Mf in their skin. Although relatively high among individual aged 21 to 30 years old, the prevalence of *O*. *volvulus* Mf was similar among the different age classes including the age class of children under 5 years, not eligible for IVM treatment (χ2: 6.285; df: 7: p-value = 0.5069). Furthermore, the comparison of Mf prevalence between children younger than 10 years (the age range recommended by the WHO for seroprevalence surveillance) and individuals aged 10 years and over showed no significantly difference (χ2: 0.03900; df: 1: p-value = 0.8435). Similarly, no difference was found when comparing Mf prevalence between male and female (χ2: 0.2821; df: 1: p-value = 0.5953) (Table 2).

**Table 2:** Onchocerciasis microfilaridermia and anti-Ov16 IgG4 prevalences, and agreement between skin snip and SD Ov16 ELISA according to age, gender and IVM intake.

	Examined	Skin Microfilariae		Anti-Ov16 Antibodies		Agreements	
		Skin Mf+	Prevalence (%)	Ov16+	Prevalence (%)	PPA (%)	PNA (%)
**Age classes (1)**							
[2–4]	63	8	12.7	14	22.2	75	85.5
[5–10]	144	39	27.1	66	45.8	84.6	68.6
[11–20]	29	8	27.6	19	65.5	87.5	42.9
[21–30]	16	6	37.5	14	87.5	83.3	10
[31–40]	18	2	11.1	15	83.3	100	18.8
[41–50]	21	5	23.8	16	76.2	100	31.3
[51–60]	28	5	17.9	20	71.4	80	30.4
[61-over]	23	7	30.4	18	78.3	52	12.5
**Age classes (2)**							
Under 10 years	188	43	22.9	72	38.3	83.7	75.2
10 years and over	154	37	24	110	71.4	81.1	31.6
**Gender**							
Males	164	41	25.0	93	56.7	82.9	52
Females	179	39	21.8	89	49.7	82.1	59
**History of IVM**							
Naïve to IVM	176	35	19.9	68	38.6	80	71.6
At Least one intake	166	45	27.1	114	68.7	84	37.2
**Overall**	**342**	**80**	**23,4**	**182**	**53.2**	**82.5**	**55.7**

### Ov16 seroprevalence

A total 8 and a half plates were run and after quality control (QC) testing, no plate was rejected, though seven individual samples were repeated for confirmation, thus suggesting high accuracy, consistency and reliability of our data. Ov16 seroprevalence was 53.2% (95%CI: 47.9–58.4), that is 182 out of the 342 individuals examined tested positive. As for Mf prevalence, individuals aged 21 to 30 years old exhibited the highest Ov16 seroprevalence. An increasing trend in Ov16 seroprevalence with age was observed until 30 years old (OR: 1.0357; 95%CI: 1.0229–1.0487, p-value<0.0001). In addition, Ov16 seroprevalence was significantly different among the different age classes (χ2: 18.09; df: 7: p-value = 0.0116), with children under 5 years (not eligible for IVM MDA) having the lowest seroprevalence. Similarly, children aged <10 years were less exposed to *O*. *volvulus* infection (38.2%) than individuals aged ≥10 years (71.4%) (χ2: 11.28; df: 1; p-value: 0.0008). On the contrary, Ov16 seroprevalence was similar in males and females (χ2: 0.4703; df: 1; p-value: 0.4928) (Table 2).

### Distribution of skin Mf counts and anti-Ov16 IgG4 in the study population

#### Distribution of skin Mf counts in the study population

**Fig 1:**
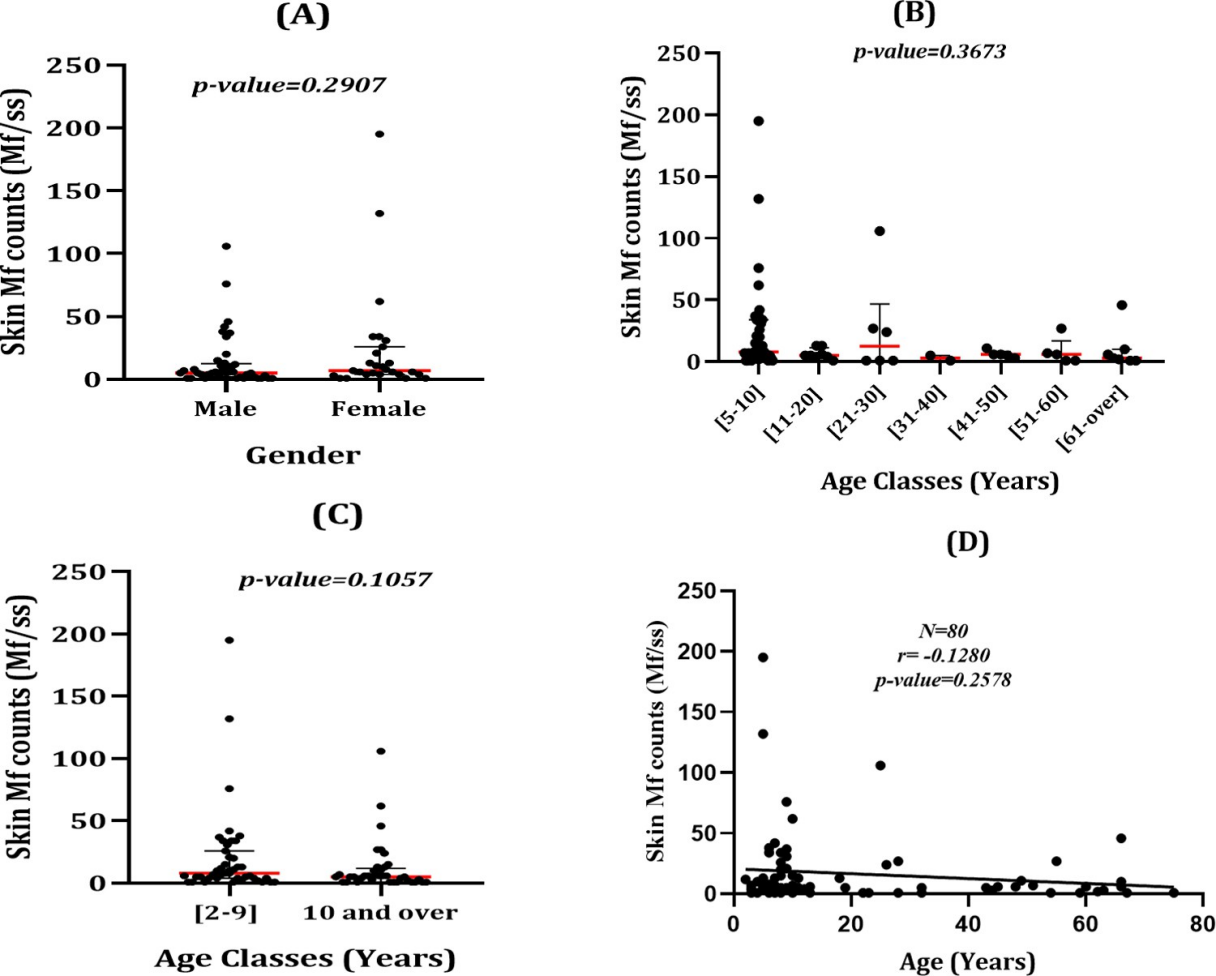
Distribution of skin Mf according to gender and age classes. (A) Distribution of skin Mf between males and females; (B) Distribution of skin Mf within the different age classes; (C) distribution of skin Mf counts between children younger than 10 and individuals aged 10 years and over; (D) Correlation between skin Mf counts and age.

Skin Mf counts (in Mf per skin snip [Mf/ss]) was widely distributed in the study population ranging from 0.5 to 195 Mf/ss (arithmetic mean: 16.7: standard deviation (SD): 30.5). Skin Mf count was compared between gender, and between different ages classes, considering children under 5 years not eligible for IVM treatment as a separate group. The median skin Mf counts was similar between males and females (Mann Whitney U: 469; p-value: 0.2907) (Fig 1A), between the difference age classes (Kruskal Wallis statistic: 7.620; p-value: 0.3673) (Fig 1B), as well as between children aged <10 years and individuals aged ≥10 years (Mann Whitney U: 628.6; p-value: 0.1057) (Fig 1C). Spearman ranked correlation test revealed no correlation between skin Mf counts and age of participants (r = 0.1280; 95%CI: -0.3441–0.1010; p-value = 0.2578) (Fig 1D).

#### Distribution of the estimated anti-Ov16 IgG4 concentration in the entire study population

**Fig 2:**
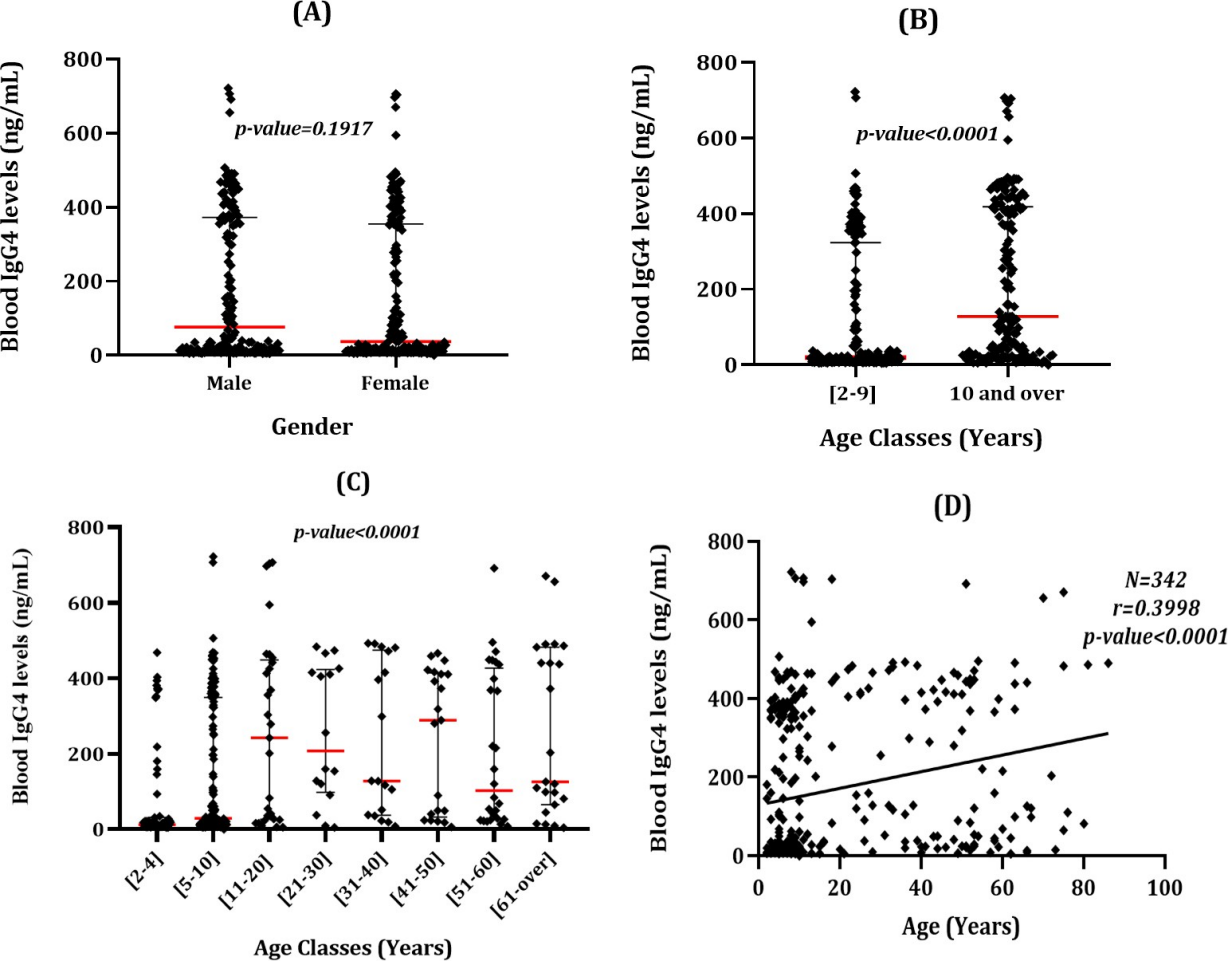
Distribution of IgG4 levels in the study population according to gender and age classes. (A) Distribution of IgG4 between males and females. (B) Distribution of IgG4 between individuals aged <10 and those aged 10 years and over; (C) Distribution of IgG4 levels within the different age classes; (D) Correlation between IgG4 levels and age. IgG4 levels were determined using the normalized OD.

Regarding Ov16 serology, the normalized optical density (OD) values varied from 0.0 to 9.680 (Arithmetic mean: 2.297; SD: 2.596), while the estimated anti-Ov16 IgG4 concentration ranged from 0.00 to 722.39 ng/mL (arithmetic mean: 171.4; SD: 193.7). This unexpected OD value (OD>4.000) is the result of data normalization that for this method takes into account the value of the calibrator provided by the manufacturer. Contrarily to skin Mf counts, high heterogeneity was observed in the distribution of the IgG4 levels in the study population. Indeed, the maximum IgG4 levels was reached in individuals aged 11–20 years old (Fig 2C). In addition, the median IgG4 was significantly different between age classes (Kruskal Wallis statistic: 58.79; p-value<0.0001). However, the Dunn’s post-hoc test revealed that only the IgG4 levels among enrollees aged 2–4 years of age was significantly lower compared to those of other age classes. A similar trend was observed in the median IgG4 of children aged <10 years old and individuals aged ≥10 years (Mann Whitney U: 8,594; p-value<0.0001) (Fig 2B). Furthermore, a positive correlation was found between IgG4 levels and age of enrollees (Spearman ranked correlation test: r = 0.4020; 95%CI = 0.3–0.5; p-value<0.0001), indicative of an increase in the anti-Ov16 IgG4 with age in this population (Fig 2D). Finally, the median IgG4 levels was similar between males and females (Mann Whitney U: 13,369; p-value: 0.2) (Fig 2A). Similar results were found when performing the analyses using OD in place of estimated IgG4 concentration (see supporting information S1 Fig).

#### Impact of IVM intake on *Ov* skin Mf counts and estimated anti-Ov16 IgG4 concentration

**Fig 3:**
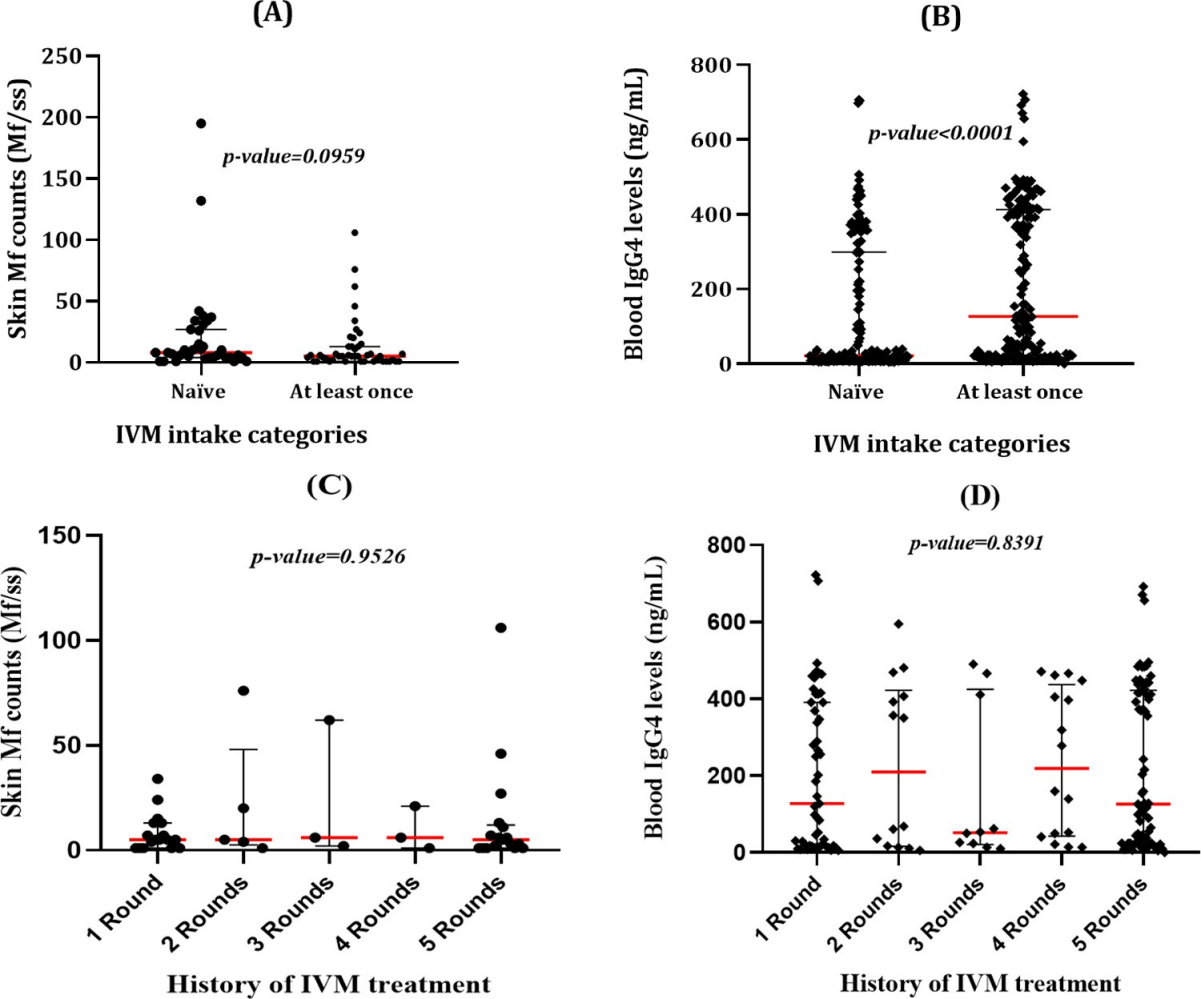
Distribution of Mf counts and IgG4 concentration according to IVM intake history. (A) Comparison of skin Mf between IVM naïve and individuals who have taken IVM at least once. (B) Comparison of IgG4 concentration between IVM naïve and individuals who have taken IVM at least once. (C) Comparison of IgG4 concentration according to the number of rounds IVM received during the last 5 years. (D) Comparison of skin Mf according to the number of rounds IVM received during the last 5 years.

To assess the impact of IVM intake on Mf counts, the latter were compared between IVM naïve individuals and those who have taken IVM at least once during the last 5 years. No significant difference was found in the median Mf counts of naïve and regularly treated individuals (Mann Whitney U: 616.5; p-value: 0.0959) (Fig 3A). In addition, no significant difference was found when comparing Mf counts according to the number of treatments received during the last 5 years (Kruskal Wallis statistics: 0.6894; p-value: 0.8376) (Fig 3D).

Regarding the impact of IVM intake on the estimated IgG4 antibody concentration, the estimated IgG4 concentration of IVM naïve individuals was significantly lower than that of individuals who have taken at least one IVM treatment during the past five years (Mann Whitney U: 9,459; p-value<0.0001) (Fig 3B), though no significant difference was found when comparing IgG4 as per the number of treatment received during the last 5 years (Kruskal Wallis statistics: 1.438; p-value = 0.8376) (Fig 3C).

#### Relationship between skin Mf counts and IgG4 levels

**Fig 4:**
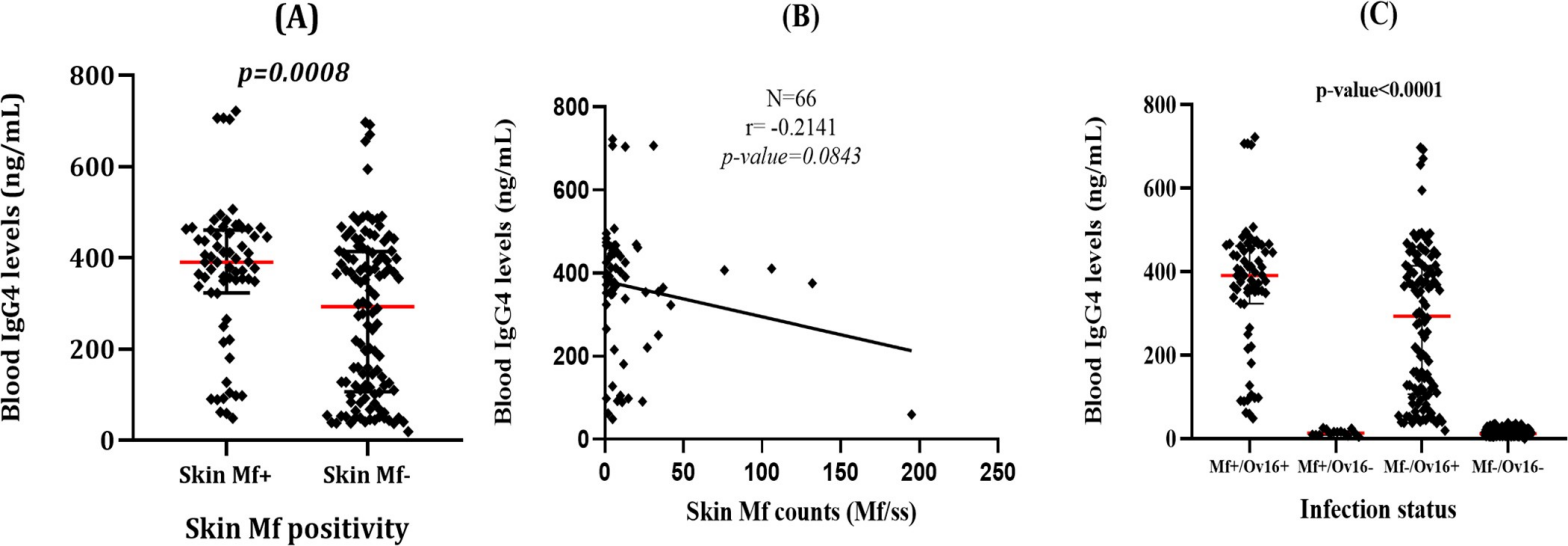
Relationship between skin microfilariae and anti-Ov16 IgG4 levels. (A) Comparison of the anti-Ov16 IgG4 levels between Mf positive and negative individuals; (B) correlation between skin Mf counts and the anti-Ov16 IgG4 levels. IgG4 levels were determined using the normalized OD.

The median IgG4 levels of individuals harboring *O*. *volvulus* Mf in their skin was significantly higher than that of those with negative skin biopsies (Mann Whitney U: 2689; p-value = 0.0008) (Fig 4A); the same trend was observed while considering only children aged <10 years old (Mann Whitney U: 1017; p-value<0.0001). Moreover, a negative correlation between skin Mf count and anti- Ov16 IgG4 levels was found using Spearman ranked correlation test (r = -0.2400; 95%CI: -0.4421–0.01474; p-value = 0.0320) (Fig 4B). However, no significant correlation was found when considering only children younger than 10 years of age (r = -0.03459; 95%CI: -0.3397–0.2771; p- value = 0.8257). Performing the same analyses using OD in place of estimated IgG4 concentration, a similar trend was found (see supporting information S1 Fig).

## Discussion

IVM-based PCT is ongoing in some onchocerciasis endemic areas since almost three decades [18]. Monitoring and evaluation of the efficacy of this strategy, and eventually the evaluation of transmission interruption requires diagnostic tools with high reliability. WHO therefore strongly recommended the use of Ov16 serology in children aged <10 years for stopping MDA, but with low evidence of certainty. To contribute evidences for the use Ov16 as evaluation tools, this study aimed at investigating *Ov* microfilaridermia and IgG4 antibodies levels to the *Ov* specific antigen Ov16 in an area where more than 20 rounds of IVM MDA have been organized. We determined and compared anti-Ov16 antibodies and skin Mf positivity, and further estimated anti-Ov16 IgG4 concentration and evaluated its variation according to IVM intake history, age, gender and Mf counts of enrollees.

During this study, a fair agreement was found between skin snip and Ov16 serology. An individual carrying *Ov* Mf in his skin had 82.5% of chances to be diagnosed positive by Ov16 serology while an Mf negative individual had 55.7% of chances to be diagnosed negative by Ov16 serology, indicative of a high positive agreement and a low negative agreement between the two tests. The PPA obtained between the two tests in this study is not quite surprising since any Ov16-based serology is assumed to systematically fail in detecting about 20% of infected individuals [7, 9], and as such the PPA between Ov16 serology and any other onchocerciasis test should be around 80%. Besides this, our results also showed that Ov16 ELISA has only 55% of chances to find a skin snip negative individual. This was also expected and can be explained by two hypotheses. First, the sensitivity of skin snip is also low. Indeed, skip biopsies could also fail in taking Mf present in the skin. Further microscopic examination of skin biopsies does not detect all Mf present in the skin biopsies [19, 20] and no further molecular analysis was done on skin biopsies in this study (especially when our study area in under repetitive MDA since >20 years). Therefore, the skin Mf positivity rate obtained here could also be underestimated. Another explanation of this low NPA between skin Mf and Ov16 serology could be that the study was conducted in the Mbam valley, an area where the disease is persisting despite long term treatment, with high prevalence and low Mf intensity. As such, Ov16 seroprevalence obtained in this study might represent cumulative infection. Regarding children aged <10 years old, that is the age class recommended for seroprevalence surveillance [3], the proportion of skin snip+/Ov16- individuals was lower (compare to that of the adult population), and a higher positive and negative agreements were found between Ov16 ELISA and skin snip. This is not surprising since, on the contrary of older individuals whose worms are became less fertile as a result of multiple rounds IVM treatment [21], worm harbored by children are fully productive and hence the better agreements between skin snip and Ov16 serology among this age class.

Mathematical modelling has established a sex- and age-profile for helminth infections. According to those models, prevalence and intensity of infection increase with age and gender, and intensity of infection is higher in male than in female [22–24], with a leveling up of Mf counts observed between 20 to 40 years of age [25, 26]. Contrariwise, no variation of Ov Mf prevalence and skin Mf counts with age and gender was observed in this study. This might be due to the fact that previously described models were done during the early phases of implementation of PCT strategies and did not take into account variations following treatments. Therefore, the difference observed in this study might be the result of IVM PCT ongoing in the area since more than two decades. However, the high proportion of non-compliance to IVM PCT found during this study might sustain a strong transmission as already suggested [27, 28], and might further explained the similar distribution of the infection among the different age classes. Also, and as expected [29], Ov16 antibodies positivity increase with age, to reach its maximum proportion in individuals aged 21 to 30 years old. This increasing trend in Ov16 seroprevalence with age was already suggested [30], and might be explained by the fact that individuals tested positive by Ov16 are those already infected by the time of implementation of control strategies (hence no significant difference was found comparing seroprevalence in the different age classes of individuals aged >20). Therefore, this persistence of high Ov16 seroprevalence might be an overestimation of the real disease prevalence. In fact, the levels of antibody to Ov16 slowly decrease after treatment [25, 31] and although there is no evidence on how long IgG4 antibodies response to Ov16 persist in exposed individuals, a persistence of circulating antigens up to seven years after parasites clearance has been suggested for lymphatic filariasis [32, 33]. These observations suggest that PCT might alter parasitological indicator of *Ov* infection but not immunological indicators. Given that, seroprevalence evaluation in individuals born before or during the first years of implementation of PCT might overestimate disease prevalence.

The microfilaricidal effect and the long-term impact of IVM on adult worm is well documented [21]. However, it is still poorly known what effect IVM has on antibodies’ response to the *Ov* specific antigen Ov16. Findings from this study showed no difference in Mf counts between IVM naïve individuals and those who have been regularly treated during the past five years. This can be explained by the fact that samples were collected ten months after the last IVM-based MDA round thus leaving enough time for recolonization of participants’ dermis with Mf or to be re-infected by those not adhering to IVM MDA, in particular in the context of very high transmission. Besides this, IVM naïve individuals had significantly low anti-Ov16 antibodies concentration than those reported having taken IVM at least once. This result is supportive of the fact that IVM intake might have no direct impact on anti-Ov16 IgG4 (and therefore Ov16 serology) [25, 31]. This finding further indicates that young children might produce less anti-Ov16 IgG4 than their older counterparts since IVM naïve individuals in this study were composed at 36% of children under 5 years not eligible for IVM treatment. Further investigations of the impact of IVM treatment on the performance of Ov16 serology are therefore needed in areas with contrasting history of IVM MDA and contrasting endemicity.

Another important finding of this study was the variation of the IgG4 levels with microfilariae counts. The fact that individuals positive for skin snip had higher IgG4 levels than their negative counterparts align with the finding from studies on animal models [11] and might suggest a positive correlation between skin Mf counts and IgG4 levels. However, a weakly negative correlation was found between skin Mf counts and IgG4 levels in this study. It was demonstrated that *O*. *volvulus* prepatent period is generally seven to twelve months, but this process can last up to three years [34]. It can therefore be thought that the high levels of anti-Ov16 antibodies in individuals with low Mf count might be due the carriage of parasites that are not yet productive or that have become less productive as a result of IVM treatment [21]. Consequently, assays detecting Ov adult parasites signature might be more reliable to assess transmission interruption than larvae-based assay.

On the opposite of skin Mf counts that were similar among the different age classes, the IgG4 levels increased with age, children younger than 5 years of age exhibiting a significantly lower IgG4 levels than their older counterparts. There are two possible explanations to this. First, the lowest Ov16 antibodies levels found in young children might be due to their low exposure to *Simulium* bites as a result of their habits/activities that can be reinforced by repeated IVM MDA ongoing in the area. Indeed, adult population already carriers of *Ov* parasites are less likely to received new infective bites (given that when infected patient ages and develops a larger total worm load, their body becomes less susceptible to infection with new blackfly-transmitted L3 larvae) [35, 36], as such, the antibodies levels might increase not as a consequence of a constant exposure but as an accumulation of adult worms. Hence the low PPA and NPA found between skin snip and Ov16 serology (NPA = 31%) in individuals aged ≥10. This hypothesis is further supported by the fact that anti- Ov16 antibodies levels was almost the same among the different age classes of individuals aged ≥10 years. Alternatively, and as suggested by modeling studies (Ov16 seroprevalence in children aged 0–4 years might generally be too close to zero) [29] children aged 2 to 4 years even if infected might take more time to mount a strong immune response to *Onchocerca volvulus* antigens [31, 33]. Considering that the age class recommended for stopping MDA using Ov16 serology include this group of children and even those younger, this calls for more investigations to refine the age class for serological surveillance.

## Limitations

A number of limitations have been identified in this study and deserve to be taken into account in further studies. (i) SD Ov16 ELISA is a qualitative ELISA method. The standard curve used to estimate Ab concentration consists of high-binding monoclonal antibodies, while antibodies in individuals might be variable. Therefore, the observations made in this study might not be similar to that of a quantitative antigen binding assay. In addition, these findings were made using SD Ov16 ELISA Kit which might be different to those of other Ov16 ELISA protocols. (ii) Only three concentrations were used to determine the standard slope, thus reducing the accuracy of the predictions. (iii) As a consequence of potentially high variation of antibody levels at individual level, longitudinal studies rather than cross-sectional studies as it was the case in the current study may be better suited for in-depth analysis of Ab levels. (iv) It is also worth to mention that although the sample size used in this study was representative of the Bafia health district, the representativeness of all the age classes considered in this study was not warranted and the stratification of age groups might bear some bias. (v) Finally, the low sensitivity of skin snip and the potential false negative for skin Mf, could influence the agreement between positivity to skin Mf and anti-Ov16 IgG4.

## Conclusion

This study reports poor agreement between parasitological (skin Mf) and immunological (Ov16 antibodies level) indicators of *Ov* infection after several rounds IVM treatment. Although our study was exploratory, our data revealed an increasing trend in antibodies’ level to Ov16 with age, as an indication of exposure and cumulative infection, and provides evidence supporting the use of Ov16 serology testing in children <10 years of age for assessment of transmission interruption. Nonetheless, the very low anti-Ov16 antibodies level observed in children aged 2 to 4 years old raises question about the inclusion of children under 5 years in the recommended age class for stopping MDA decision-making, especially because the blood drawing process is somehow painful and invasive. More investigations are needed on the antibody response to Ov16 in the <5 years age class.

## Supporting information

S1 Fig Distribution of normalized optical density (OD) in the study population according to gender and age classes. (A) Distribution of normalized OD between males and females. (B) Distribution of normalized OD between individuals aged <10 and those aged 10 years and over; (C) Distribution of normalized OD within the different age classes; (D) Correlation between normalized OD and age.
(TIF)

S2 Fig Relationship between skin microfilariae and normalized optical density (OD). (A) Comparison of normalized OD between Mf positive and negative individuals; (B) correlation between skin Mf counts and normalized OD.
(TIF)

S1 Text Procedure for estimation of IgG4 concentrations and comparison of the optical densities between the different covariates. (DOCX)
