# Supplementary figures and images for: Relationship between skin snip and Ov16 ELISA: Two diagnostic tools for onchocerciasis in a focus in Cameroon after two decades of ivermectin-based preventive chemotherapy
Source: PLoS Negl Trop Dis. 2022 May 2;16(5):e0010380. doi: 10.1371/journal.pntd.0010380 (PMC9098087; doi:10.1371/journal.pntd.0010380)

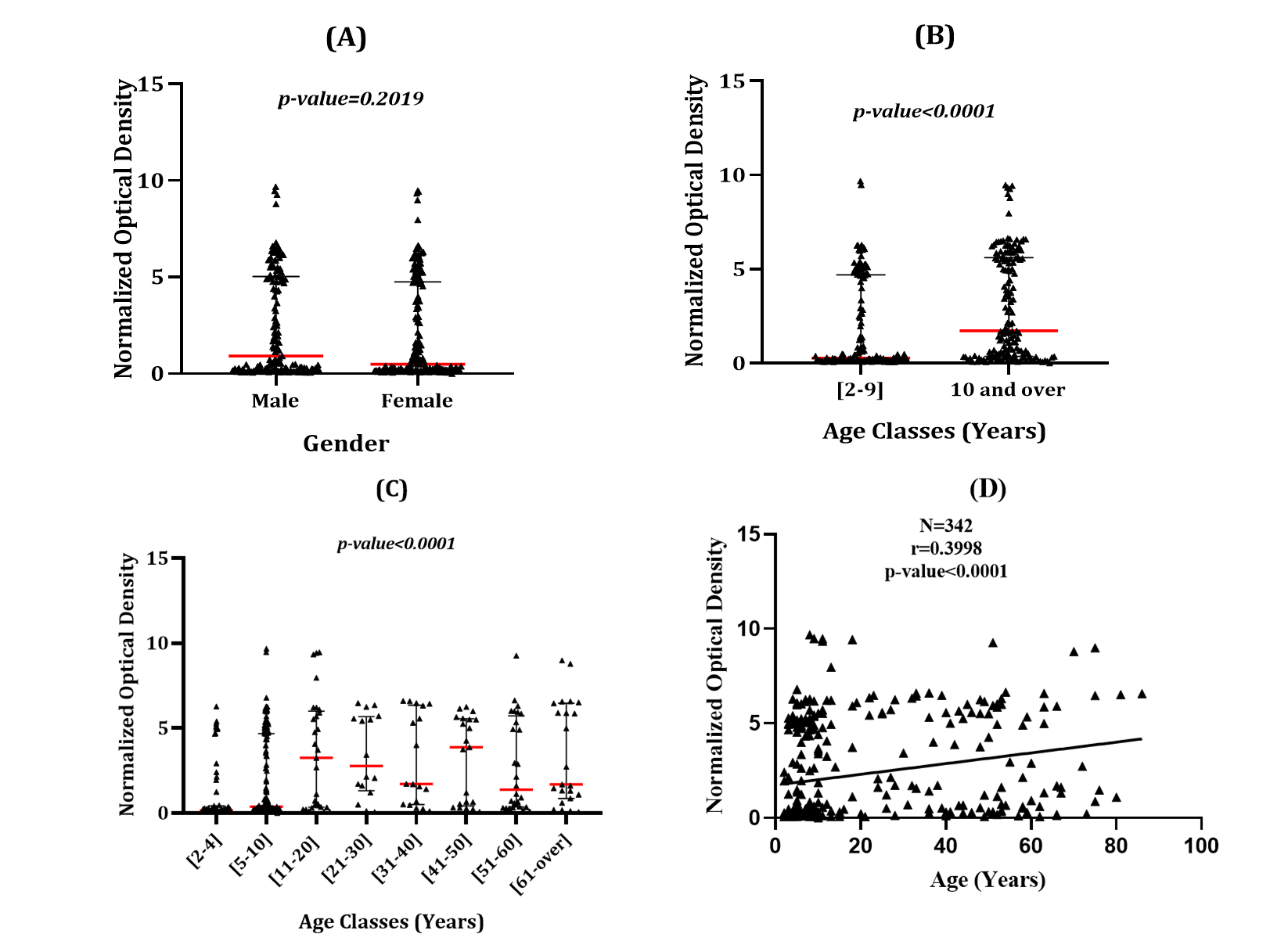

Supplement: S1 Fig — (A) Distribution of normalized OD between males and females. (B) Distribution of normalized OD between individuals aged <10 and those aged 10 years and over; (C) Distribution of normalized OD within the different age classes; (D) Correlation between normalized OD and age. (TIF) [file pntd.0010380.s001.tif]

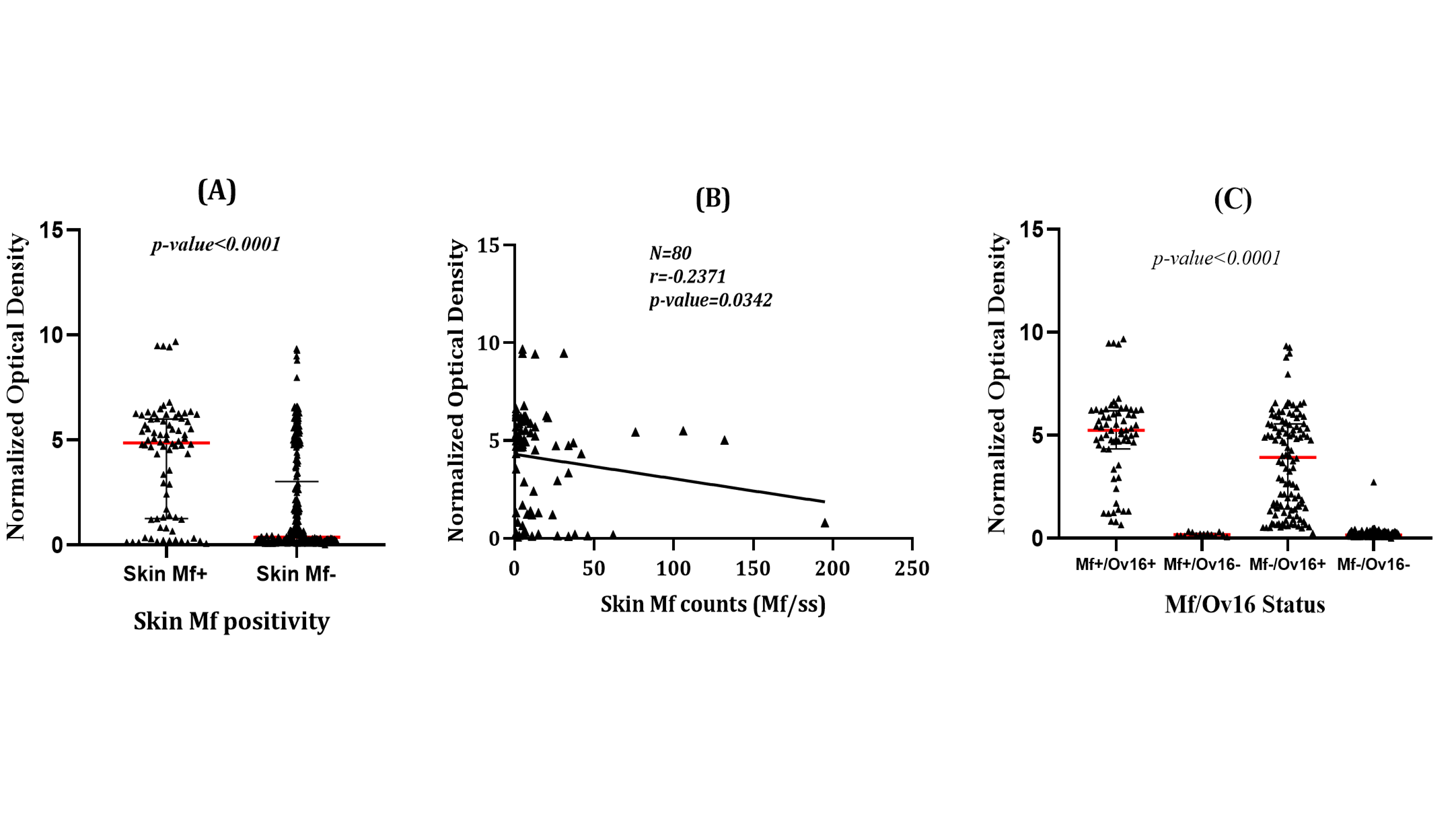

Supplement: S2 Fig — (A) Comparison of normalized OD between Mf positive and negative individuals; (B) correlation between skin Mf counts and normalized OD. (TIF) [file pntd.0010380.s002.tif]
