# Supplementary material for: Relationship between skin snip and Ov16 ELISA: Two diagnostic tools for onchocerciasis in a focus in Cameroon after two decades of ivermectin-based preventive chemotherapy
Source: PLoS Negl Trop Dis. 2022 May 2;16(5):e0010380. doi: 10.1371/journal.pntd.0010380 (PMC9098087; doi:10.1371/journal.pntd.0010380)
Supplement: S1 Text — (DOCX) [file pntd.0010380.s003.docx]

**S1 File. Procedure for estimation of IgG4 concentrations and comparison of the optical densities between the different covariates. (DOCX)**

**Estimation of IgG4 concentrations**

Recombinant AbD19432 Ab at three different concentrations (200, 50 and 25 ng/ml, diluted to 1/50) were used for each plate to estimate anti-Ov16 IgG4 antibody concentration. Given that a total of 8 plates were processed, 8 different ODs were available for each standard. The mean OD for standards was calculated for the total number of plates (see the Table below). Using those OD values, a calibrating curve was drawn with the concentration in Ab on x-axis and the OD value on y-axis (see the Figure below). The estimated Ab concentration for each sample was then determined (for all the study participant) using the following formula, the dilution factor being 0.02 and the concentration expressed in ng/ml.

$$Ab concentation =\frac{Sample OD}{Slope}*Dilution factor$$

| Plate | 4ng | 1ng | 0,5ng |
| --- | --- | --- | --- |
| Plate1 | 2,528 | 0,936 | 0,470 |
| Plate2 | 2,818 | 0,957 | 0,510 |
| Plate3 | 2,995 | 1,066 | 0,544 |
| Plate4 | 3,633 | 1,385 | 0,840 |
| Plate5 | 3,173 | 1,111 | 0,568 |
| Plate6 | 3,972 | 1,305 | 0,644 |
| Plate7 | 3,139 | 0,957 | 0,541 |
| Plate8 | 3,203 | 1,012 | 0,556 |
| Mean | **2,782** | **0,965** | **0,515** |

The table below presents the computation of mean OD value for standards

The figure below is the calibrating curve with the concentration in Ab on x-axis and the OD value on y-axis.

From the figure above, the linear equation is y=0.6731+0.2598. The slope being 0.6731 and the dilution factor 0.02, the Ab concentration is finally computed as follows:

$$Ab concentation =\frac{Sample OD}{0.6731}*0.02= \frac{Sample OD}{0.0134}$$

**Comparison of the optical densities between the different groups**

The highest OD value (median value) is observed among individuals aged 11-20 years old (S1 Fig C). The median OD was significantly different between age classes (Chi-square: 57.47; p-value<0.0001). Additionally, the Dunn’s post-hoc test revealed that the OD values of enrollees aged 2-4 years was significantly lower compared to those of other age classes. A similar trend was observed for the median OD of children younger than 10 years old and their older counterparts (Mann Whitney U: 8,032; p-value <0.0001) (S1 Fig B). Furthermore, a positive correlation was found between OD values and age of enrollees (Spearman ranked correlation test: r=03998; 95%CI=0.3039-0.4877; p-value<0.0001), indicative of an increase in OD value with age in this population (S1 Fig D). Finally, the median OD was similar between males and females (Mann Whitney U: 13,396; p-value=0.2019) (S1 Fig A).

Besides all the above, the median OD value of individuals harboring *O. volvulus* Mf in their skin was significantly higher than that of Mf negative individuals (Mann Whitney U: 5,310; p-value <0.0001) (S2 Fig A). Moreover, a negative correlation was found between OD and skin Mf counts (Spearman ranked correlation test: r=-0.2371; 95%CI=-0.4396 to -0.01173; p-value=0.0342) (S2 Fig B).
